# An ecometric analysis of neighbourhood cohesion

**DOI:** 10.1186/1478-7954-4-17

**Published:** 2006-12-21

**Authors:** David L Fone, Daniel M Farewell, Frank D Dunstan

**Affiliations:** 1 Department of Epidemiology, Statistics and Public Health, Centre for Health Sciences Research, School of Medicine, Cardiff University, Neuadd Meirionnydd, Heath Park, Cardiff, Wales, CF14 4YS, UK; 2 National Public Health Service for Wales, Mamhilad Park Estate, Pontypool, Torfaen, Wales, NP4 0YP, UK

## Abstract

**Background:**

It is widely believed that the social environment has an important influence on health, but there is less certainty about how to measure specific factors within the social environment that could link the neighbourhood of residence to a health outcome. The objectives of the study were to examine the underlying constructs captured by an adapted version of Buckner's neighbourhood cohesion scale, and to assess the reliability of the scale at the small-area-level by combining ecometric methodology with ordinal modelling of a five-point scale.

**Methods:**

Data were analysed from 11,078 participants in the Caerphilly Health and Social Needs Study, who were sampled from within 325 UK census enumeration districts in Caerphilly county borough, Wales, UK. The responses of interest came from 15 question items designed to capture different facets of neighbourhood cohesion. Factor analysis was used to identify constructs underlying the neighbourhood cohesion item responses. Using a multilevel ecometric model, the variability present in these ordinal responses was decomposed into contextual, compositional, item-level and residual components.

**Results:**

Two constructs labelled neighbourhood belonging and social cohesion were identified, and variability in both constructs was modelled at each level of the multilevel structure. The intra-neighbourhood correlations were 6.4% and 1.0% for the neighbourhood belonging and social cohesion subscales, respectively. Given the large sample size, contextual neighbourhood cohesion scores can be estimated reliably. The wide variation in the observed frequency of occurence of the scale item activities suggests that the two subscales have desirable ecometric properties. Further, the majority of between-neighbourhood variation is not explained by the socio-demographic characteristics of the individual respondents.

**Conclusion:**

Assessment of the properties of the adapted neighbourhood cohesion scale using factor analysis and ecometric analysis extended to an ordinal scale has shown that the items allow fine discrimination between individuals. However, large sample sizes are needed in order to accurately estimate contextual neighbourhood cohesion. The scale is therefore appropriate for use in the measurement of neighbourhood cohesion at small-area-level in future studies of neighbourhoods and health.

## Background

In recent years there has been an increasing level of interest in researching neighbourhood effects on health [1]. It is widely believed that the social environment has an important influence on health and well-being [2], but it is less certain how to conceptualise, define, operationalise and measure specific factors and pathways within the social environment that link the neighbourhood of residence to health outcome [3]. One aspect of the social environment in which there has been much interest in recent years is the concept of social capital. Putnam defines social capital as "features of social organisation, such as trust, norms, and networks, that can improve the efficiency of society by facilitating coordinated actions" [4]. Although several studies have suggested a beneficial effect of social capital on various measures of health [5], there is a long-standing debate in the literature on the concepts and measurement of social capital [6], and still a lack of agreement on whether social capital is a function of individuals and their social interactions within social networks or whether it is a collective attribute of communities and societies [7]. As Kawachi *et al*. [7] argue, however, this may be a false dichotomy. Social capital should be measured and analysed in empirical studies of social capital and health at both individual and contextual levels in a multilevel framework, so that joint individual- and group-level mechanisms can be explored [8].

A wide range of social capital indicators have been developed, some of which flow from the twin-concept model of 'cognitive' social capital, measured by perceived levels of support, reciprocity, sharing and trust, and 'structural' social capital, which includes the extent and intensity of associational links [9]. The problem of measurement remains. In this paper we focus on the measurement of neighbourhood cohesion as a measure of cognitive and structural social capital, using the neighbourhood cohesion scale developed originally by Buckner [10]. Potential mechanisms for how neighbourhood cohesion might affect health outcomes have been empirically tested in a study of community attachment, showing that residential stability has positive individual and contextual effects on local friendship ties, collective attachment, and rates of local social participation [11].

Following psychometric analysis, Buckner's final Neighbourhood Cohesion scale included 18 question items. The scale was subsequently validated in a Canadian study, after reducing to 17 items [12]. In the UK, the scale has been further adapted for community studies of neighbourhoods and health [13,14], and eight items from the scale have been used in the British Household Panel Survey (BHPS) as a measure of 'neighbourhood attachment' [15,16]. Although the Neighbourhood Cohesion scale was also intended by Buckner for use as a group-level measure by aggregation of individual-level responses to calculate an area mean score [10], none of these studies [13-16] attempted a contextual measure of neighbourhood cohesion using survey responses. Community-based surveys can give valid and reliable measures of neighbourhood social processes [1], but before aggregate area measures are used, a newly described methodology to assess the properties of a scale at the ecological level – the science of 'ecometrics' [17] – should be followed. In assessing a social capital scale, two scientific principles must be borne in mind. First, Rasch [18] has pointed out that data resulting from subjects answering questions are *comparisons *rather than *measurements*: comparing the 'neighbourliness' of an individual to the 'distinctiveness' of behaviour implied by a specific question item. A neighbourly individual will be more likely to exhibit distinctive (positive) behaviour than an unneighbourly individual. Thus a response to a questionnaire should be understood as being composed of effects due to the particular question (its distinctiveness), the individual respondent (their neighbourliness) and, by extension, the social and spatial context of this respondent – the 'cohesion' of their neighbourhood. This point is discussed in some detail by Tennant *et al*. [19]. Secondly, Raudenbush and Sampson [17] argue that any suitable scale will be based on enquiries about behaviour patterns whose distinctivenesses vary substantially. A questionnaire designed to elicit information about social capital, for example, should fill the spectrum from the commonplace to the rare, in order to sharply differentiate between individuals, and between communities.

Ecometrics is a novel approach to the assessment of neighbourhoods, though as Gauvin *et al*. [20] point out, it is essentially an integration of item-response theory into that of hierarchical modelling. Echeverria *et al*. [21] argue that, following a single-level reliability analysis, ecometrics is a logical "next step in the evaluation of the utility of self-reported neighborhood characteristics". Multilevel methods decompose the variation present in the data into a hierarchy of sources: contextual, individual, item and residual. In particular, this allows us to decide if the variability in area-level measures of neighbourhood cohesion is chiefly a function of the neighbourhoods that compose them, the individuals therein, both or neither. Another advantage of the multilevel approach to analysis of neighbourhood cohesion scores is the ease with which reliability may be assessed; following [18], it is the *latent *cohesion that should be measured reliably, rather than the comparison of that cohesion with a particular question.

Many instances of ecometrics in the literature have shared the same application area with Raudenbush and Sampson [17], namely the evaluation of the physical properties of neighbourhoods [20,22,23]. In this paper we investigate the measurement of individual and small area-level neighbourhood cohesion using an ecometric analysis of an adapted version of the Buckner Neighbourhood Cohesion scale [10]. We have gathered in-depth geographically referenced and representative survey data from over 12,000 adult residents of Caerphilly county borough, a region of south-east Wales in the UK. The data come from the Caerphilly Health and Social Needs Study, a community study of health and social inequality set in a deprived post-industrial area of Wales [24-26]. Caerphilly county borough is one of the 22 local government areas in Wales created in 1996 as part of the reorganisation of local government and is one of the five unitary authorities situated within the former Gwent Health Authority area. The borough occupies 28,000 hectares of the South Wales valleys, between the urban centres of Cardiff and Newport in the south and the Brecon Beacons to the north, with a declining and ageing population of 169,519 (2001 Census). The specific objectives of this paper are, firstly, to assess the underlying constructs captured by the adapted Neighbourhood Cohesion scale and, secondly, to assess the reliability of the adapted Neighbourhood Cohesion scale measured at the 1991 Census enumeration district small area-level, by adapting the ecometric methods of Raudenbush and Sampson [17]. To do so, we combine their multilevel analysis with an ordinal model for a five-point Likert scale.

## Methods

### Population survey

In autumn 2001 we carried out a cross-sectional postal questionnaire survey of the adult population aged 18 years and over resident in Caerphilly County Borough, Wales, UK. The survey was granted ethical approval by Gwent Local Research Ethics Committee and is described in detail elsewhere [25,26]. In brief, we obtained a representative dataset on 12,092 individuals linked by postcode to one of 325 enumeration districts defined by the 1991 Census in Caerphilly borough. Stratifying by electoral wards, individuals were sampled randomly, and the overall response rate was 63%. Enumeration districts were the most relevant second-level units since they were the smallest, most socially homogeneous, geographical area available in the UK 1991 Census. The mean enumeration district adult population was 406 and the response dataset included a mean of 37 (standard deviation 18, range 5 to 133, interquartile range 24 to 45) respondents per enumeration district.

The questionnaire included a wide range of socio-demographic and socio-economic questions including age; gender; occupational status in six categories of employed (full time or part-time), unemployed and seeking work, looking after home or children full time or a long term carer, full time student or at school or on a government training scheme, retired from paid work, or permanently unable to work due to illness or disability (labelled as 'incapacity'); Registrar General social class [27] coded in five categories of social class I&II (professional and intermediate), III non-manual (skilled non-manual), III manual (skilled manual), IV&V (semi-skilled and unskilled), and other; housing tenure, coded as owner occupier ('I own it or live with the person who owns it') or not owner occupier (rented); and gross household income in three categories of 'high', 'moderate' and 'low', according to whether the gross household income was greater than £215 per week, between £95 and £215 per week, or less than £95 per week. Both 'moderate' and 'low' categories are classified as 'poverty' under the UK definition; this identifies households with a gross income of less than 60% of median income, after housing costs [28]. Household income was trichotomised, as opposed to any finer categorisation, because it was felt that individuals were more likely to respond to a question on income in fairly broad categories. We also obtained the household council tax valuation band as a further measure of socio-economic status for each respondent, dichotomised into bands A&B (property value less than £39,000) and C-H (property value greater than £39,000) for the analysis [25].

### Neighbourhood cohesion scale

The Caerphilly Health and Social Needs Study steering group decided to adapt the Neighbourhood Cohesion scale for use in the wider study of health inequality in the borough, and to achieve comparability with a previous UK study [13]. In this version, 15 question items were asked (Table 1), reduced from the 17-item version [12] by removing three questions: 'If the people in my neighbourhood were planning something, I'd think of it as something 'we' were doing rather than 'they' were doing'; 'I think I agree with most people in my neighbourhood about what is important in life'; and 'I feel loyal to the people in my neighbourhood', and adding the item 'Overall, I think this is a good place to bring up children'. Each item gave a five-category Likert response scale, consisting of the options strongly agree, agree, neither agree or disagree, disagree and strongly disagree, scored from 5 to 1, respectively. Item 5, 'Given the opportunity, I would like to move out of this neighbourhood', and Item 12, 'I rarely have a neighbour over to my house to visit', were reverse scored for the analysis.

**Table 1:** The adapted neighbourhood cohesion scale

How much do you agree with the following statements about your neighbourhood...	
Item	Statement

1	Overall, I am attracted to living in this neighbourhood
2	I feel like I belong to this neighbourhood
3	I visit my friends in their homes
4	The friendships and associations I have with other people in my neighbourhood mean a lot to me
5	Given the opportunity, I would like to move out of this neighbourhood
6	If I need advice about something I could go to someone in my neighbourhood
7	I believe my neighbours would help in an emergency
8	I borrow things and exchange favours with my neighbours
9	I would be willing to work together with others on something to improve my neighbourhood
10	I plan to remain a resident of this neighbourhood for a number of years
11	I like to thing of myself as similar to the people who live in this neighbourhood
12	I rarely have a neighbour over to my house to visit
13	I regularly stop and talk with people in my neighbourhood
14	Living in this neighbourhood gives me a sense of community
15	Overall I think this is a good place to bring up children

The Likert response scale comprised the following five options: Strongly agree, Agree, Neither agree or disagree, Disagree, Strongly disagree.

### Factor analysis

The original scale development was based on three constructs relating to psychological sense of community, attachment to neighbourhood and neighbourhood interactions, but the reported factor analysis suggested that the final scale was unidimensional [10]. Our *a priori *intention was to investigate whether these constructs were, in our sample, identifiable in the adapted version of the neighbourhood cohesion scale. We hoped to identify subscales that were related as closely as possible to the structural and cognitive model of social capital, as summarised by Harpham [9]. As a first approximation we took individuals to be independent and responses to be continuous; both of these assumptions have the potential to introduce bias [29,30] but are convenient since they permit an analysis using standard software. Using principal component analysis to determine an appropriate number of latent constructs, we then used factor analysis followed by a varimax rotation to investigate the structure of a hypothetical set of latent variables that explain the pattern of correlations within the observations. The factor analysis was carried out in SPSS Version 11 [31].

### Ecometrics

Since a number of items were answered by each respondent, who in turn was sampled from an enumeration district (ED), a multilevel structure to data modelling was preferred. Such a structure admits the correlation which can arise due to commonality between individual responses to different items, and between individuals from the same area. The response variables were ordinal, so a generalised linear mixed model (GLMM) [32] was particularly appropriate. Note that each individual answers *the same *15 questions: we assume that individual *j *in area *i *gives an answer of *Y*_*ijk *_to item *k*. If there are *I *areas, *J*_*i *_individuals in area *i*, and *K *items making up the subscale, a GLMM for such data is given by

logit **P**(*Y*_*ijk *_= *l*|*η*_*ijk*_, *Y*_*ijk *_≥ *l*) = *θ*_*l *_+ *η*_*ijk *_    (1)

*i *= 1, ..., *I*; *j *= 1, ..., *J*_*i*_; *k *= 1, ..., *K*

where *l *ranges over the integers from 1 to 4 (there being 5 categories to each question, so that, given *Y*_*ijk *_≥ 5, necessarily *Y*_*ijk *_= 5). Here *η*_*ijk *_represents a mixture of *fixed effects*, covariates such as employment status or council tax band, and *random effects *attributed to a particular individual or area.

The mixture *η*_*ijk *_is taken to be linear, so that for example

*η*_*ijk *_= *γ*_*k *_- *X*_*ij*_*β *- *U*_*i *_- *V*_*ij *_    (2)

combines the distinctiveness *γ*_*k *_of item *k*, the effects *β *of covariates *X*_*ij *_(which may be contextual or compositional) together with an area-level deviation *U*_*i *_from the mean (area *i*'s cohesion, say) and individual deviation *V*_*ij *_(in area *i*, individual *j*'s neighbourliness, say). The choice of sign of the various terms is to aid interpretation: a large *γ*_*k *_is associated with distinctive behaviour, while large values of *β*, *U*_*i *_and *V*_*ij *_lead to increased probability of the higher ordinal categories. Because the question items are known and specific, as opposed to being drawn at random from some larger, hypothetical population, we modelled their distinctivenesses *γ*_*k *_as fixed effects, subject to the condition ∑_*k*_*γ*_*k *_= 0 on each subscale. Equations (1) and (2) define a *multilevel continuation ratio *model. It is a *continuation ratio *model [33] as it may be expressed in terms of the ratio of probabilities of not continuing, and continuing, to the next category *l *+ 1; it is a *multilevel *model because *η*_*ijk *_comprises area, individual and item effects. We note the observation in [34] that continuation ratio models can result in qualitatively the same conclusions and comparable model fits to the popular *grouped continuous *models for **P**(*Y*_*ijk *_≤ *l*|*η*_*ijk*_).

It is customary to assume that *U*_*i *_and *V*_*ij *_are independent and normally distributed, with variances σU2
 MathType@MTEF@5@5@+=feaafiart1ev1aaatCvAUfKttLearuWrP9MDH5MBPbIqV92AaeXatLxBI9gBaebbnrfifHhDYfgasaacH8akY=wiFfYdH8Gipec8Eeeu0xXdbba9frFj0=OqFfea0dXdd9vqai=hGuQ8kuc9pgc9s8qqaq=dirpe0xb9q8qiLsFr0=vr0=vr0dc8meaabaqaciaacaGaaeqabaqabeGadaaakeaaiiGacqWFdpWCdaqhaaWcbaGaemyvaufabaGaeGOmaidaaaaa@30C8@ and σV2
 MathType@MTEF@5@5@+=feaafiart1ev1aaatCvAUfKttLearuWrP9MDH5MBPbIqV92AaeXatLxBI9gBaebbnrfifHhDYfgasaacH8akY=wiFfYdH8Gipec8Eeeu0xXdbba9frFj0=OqFfea0dXdd9vqai=hGuQ8kuc9pgc9s8qqaq=dirpe0xb9q8qiLsFr0=vr0=vr0dc8meaabaqaciaacaGaaeqabaqabeGadaaakeaaiiGacqWFdpWCdaqhaaWcbaGaemOvayfabaGaeGOmaidaaaaa@30CA@, respectively. The ideals of Raudenbush and Sampson [17] may then be summarised as follows:

1. Area-level variation σU2
 MathType@MTEF@5@5@+=feaafiart1ev1aaatCvAUfKttLearuWrP9MDH5MBPbIqV92AaeXatLxBI9gBaebbnrfifHhDYfgasaacH8akY=wiFfYdH8Gipec8Eeeu0xXdbba9frFj0=OqFfea0dXdd9vqai=hGuQ8kuc9pgc9s8qqaq=dirpe0xb9q8qiLsFr0=vr0=vr0dc8meaabaqaciaacaGaaeqabaqabeGadaaakeaaiiGacqWFdpWCdaqhaaWcbaGaemyvaufabaGaeGOmaidaaaaa@30C8@ should be large if indeed areas are distinct.

2. Individual-level variation σV2
 MathType@MTEF@5@5@+=feaafiart1ev1aaatCvAUfKttLearuWrP9MDH5MBPbIqV92AaeXatLxBI9gBaebbnrfifHhDYfgasaacH8akY=wiFfYdH8Gipec8Eeeu0xXdbba9frFj0=OqFfea0dXdd9vqai=hGuQ8kuc9pgc9s8qqaq=dirpe0xb9q8qiLsFr0=vr0=vr0dc8meaabaqaciaacaGaaeqabaqabeGadaaakeaaiiGacqWFdpWCdaqhaaWcbaGaemOvayfabaGaeGOmaidaaaaa@30CA@ should be small so that different areas may be reliably distinguished.

3. The item distinctivenesses *γ*_*k *_should vary widely so that different areas may be finely distinguished.

We combined the first two of these ideals into a measure of reliability, and investigated the third ideal graphically.

Models such as (1) can be fitted and graphically explored using the lme4 [35] package for the R statistical software [36]. All exploratory and multilevel analyses were therefore carried out in R.

For both neighbourhood cohesion subscales, we began by fitting a null (covariate-free) model, before proceeding to include covariates. This allowed us to determine whether the variability in responses to neighbourhood cohesion question items could, or could not, largely be determined by the combined effects of individual and context.

## Results

### Population survey

**Figure 1:**
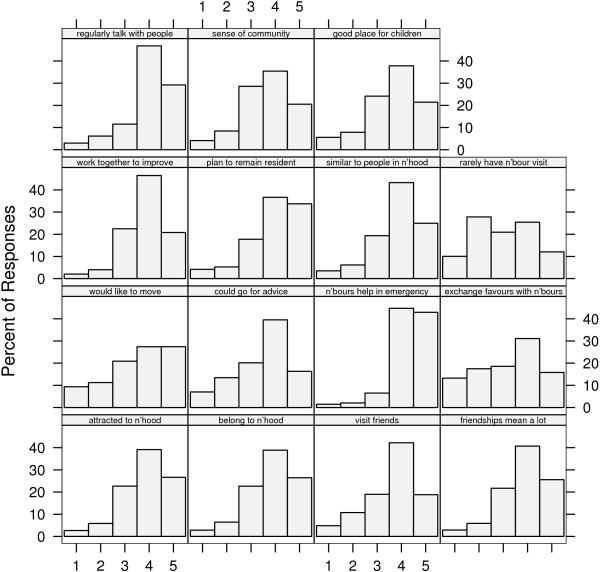
**Histogram of responses by question item**. In each instance '1' corresponds to the lowest response category of neighbourhood cohesion, and '5' to the highest category of neighbourhood cohesion. The abbreviations n'hood and n'bour(s) denote neighbourhood and neighbour(s), respectively.

Of the 12,092 respondents to the survey, we analysed data from the 11,078 (91.6%) individuals who answered all 15 stems of the Neighbourhood Cohesion scale. Figure 1 shows a histogram of the observed responses to the 15 question items. In each instance high values reflect greater neighbourhood cohesion. There are evident differences between questions: that neighbours would help in an emergency (item 7), for example, was almost universally felt, while borrowing and exchanging (item 8) was much less common. The general skew of almost all 15 items towards the more positive responses is also clear.

### Factor analysis

**Figure 2:**
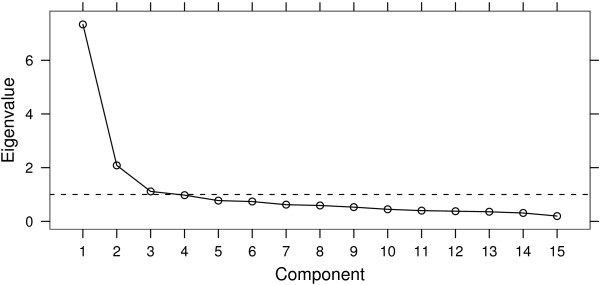
**Scree plot from Principal Components Analysis**. The eigenvalues – that is, the proportion of variance explained by each component – plotted against component number and ordered by decreasing eigenvalue.

Following a single-level principal components analysis, we produced a scree plot (Figure 2) and looked for an "elbow" in this picture. We determined that a two factor solution was an appropriate simplification of the adapted neighbourhood cohesion questionnaire items. These factors accounted respectively for 26% and 22% of the item-level variability; the factor loadings are shown in Table 2. We partitioned the items into the two factors according to the greater factor loading on individual items. In this instance, if a factor loading on a particular component exceeded 0.5, we included a question item into that component. Seven questions were included in the first component; the two largest factor loadings were for the items 1 ('Overall, I am attracted to living in this neighbourhood') and 5 ('Given the opportunity, I would like to move out of this neighbourhood', reverse coded). As these clearly related to the 'degree of attraction to the neighbourhood' originally proposed by Buckner, we labelled this component 'neighbourhood belonging'. Eight items were identified in the second component; the two largest factor loadings were for the items 8 ('I borrow things and exchange favours with my neighbours') and 4 ('The friendships and associations I have with other people in my neighbourhood mean a lot to me'). As these items clearly related to the 'degree of interaction within the neighbourhood' construct proposed by Buckner, we labelled this component 'social cohesion'. If a third principal component (which could also be supported by Figure 2) was included, it accounted for a further 6% of the variance. Examination of the factor loadings showed that this third component split the social cohesion component into two subcomponents of three and five items each, and did not identify a separate construct. We therefore considered the two component solution, shown in Table 2, to be satisfactory.

**Table 2:** Factor loadings for the two-factor solution, following a varimax rotation

Item	Factor 1 (NB)	Factor 2 (SC)	Subscale	Item-subscale correlation
1	0.846	0.133	NB	0.773
2	0.811	0.288	NB	0.793
3	0.161	0.662	SC	0.549
4	0.399	0.671	SC	0.658
5	0.815	0.094	NB	0.713
6	0.315	0.653	SC	0.596
7	0.326	0.572	SC	0.535
8	0.091	0.707	SC	0.550
9	0.119	0.536	SC	0.403
10	0.778	0.232	NB	0.740
11	0.657	0.364	NB	0.662
12	0.058	0.584	SC	0.420
13	0.336	0.522	SC	0.478
14	0.679	0.481	NB	0.728
15	0.749	0.202	NB	0.695

Also shown in the table are the subscale allocations, with NB denoting the subscale labelled 'neighbourhood belonging' and SC referring to the 'social cohesion' items, and Spearman's rank correlation coefficient between each item and the sum of the other subscale items.

We also considered the possibility that these two subscales could potentially be correlated. Upon application of a promax (oblique) rotation, the only item to change subscales was 14 ('Living in this neighbourhood gives me a sense of community'), with factor loadings 0.423 and 0.483 on the neighbourhood belonging and social cohesion subscales, respectively. Given the similarity of these values, and with reference to the rather more pronounced difference between them under varimax, we included this item as part of the neighbourhood belonging subscale.

We examined the potential for different factor structures to be operating at the different levels of the model. To do so, we used the decomposition

Yijk=Y¯i⋅k+(Yijk−Y¯i⋅k)     (3)
 MathType@MTEF@5@5@+=feaafiart1ev1aaatCvAUfKttLearuWrP9MDH5MBPbIqV92AaeXatLxBI9gBaebbnrfifHhDYfgasaacH8akY=wiFfYdH8Gipec8Eeeu0xXdbba9frFj0=OqFfea0dXdd9vqai=hGuQ8kuc9pgc9s8qqaq=dirpe0xb9q8qiLsFr0=vr0=vr0dc8meaabaqaciaacaGaaeqabaqabeGadaaakeaacqWGzbqwdaWgaaWcbaGaemyAaKMaemOAaOMaem4AaSgabeaakiabg2da9iqbdMfazzaaraWaaSbaaSqaaiabdMgaPjabgwSixlabdUgaRbqabaGccqGHRaWkcqGGOaakcqWGzbqwdaWgaaWcbaGaemyAaKMaemOAaOMaem4AaSgabeaakiabgkHiTiqbdMfazzaaraWaaSbaaSqaaiabdMgaPjabgwSixlabdUgaRbqabaGccqGGPaqkcaWLjaGaaCzcamaabmaabaGaeG4mamdacaGLOaGaayzkaaaaaa@4D1E@

to yield 325 ED-level 'responses' and a residual corresponding to each individual. At both the individual and ED levels the resulting factor structures closely matched the original, with 2 items changing subscales at the ED level and only 1 at the individual level. None of these changes represented a substantial numerical change in the factor loadings, and consequently we chose to adopt the single-level factor structure as the most parsimonious combination of these results.

It could be argued that other items, such as 15 (relating to the neighbourhood's suitability as a place to bring up children), are qualitatively different from the rest of the neighbourhood belonging subscale. We do not claim that this, or the social cohesion subscale, are in reality unidimensional constructs, and it seems likely that the degree of attraction to a neighbourhood should encapsulate only part of a wider picture. Since item 15 loads strongly onto the neighbourhood belonging subscale, we must suppose that deeming a place fit to bring up children is related to a person's sense of belonging to their neighbourhood. In accordance with Buckner's original intention, we summed the responses to the items in each subscale with equal weighting to create a neighbourhood belonging subscale with the range of possible scores from 7 to 35 and a social cohesion subscale with a range from 8 to 40. The Cronbach's alpha value for the neighbourhood belonging and social cohesion subscales were 0.908 and 0.802, respectively. The magnitude of the item-scale (Table 2) and inter-item (Tables 3 and 4) correlations suggested that both subscales achieved an acceptable degree of consistency [37].

**Table 3:** Inter-item rank correlation coefficients for neighbourhood belonging subscale

	1	2	5	10	11	14	15
1	1.000	0.753	0.651	0.602	0.501	0.546	0.633
2	0.753	1.000	0.614	0.625	0.564	0.665	0.574
5	0.651	0.614	1.000	0.647	0.483	0.521	0.546
10	0.602	0.625	0.647	1.000	0.636	0.578	0.536
11	0.501	0.564	0.483	0.636	1.000	0.597	0.497
14	0.546	0.665	0.521	0.578	0.597	1.000	0.601
15	0.633	0.574	0.546	0.536	0.497	0.601	1.000

Spearman's rank correlation coefficient between items in the neighbourhood belonging subscale, calculated using pairwise complete observations.

**Table 4:** Inter-item rank correlation coefficients for social cohesion subscale

	3	4	6	7	8	9	12	13
3	1.000	0.524	0.391	0.299	0.369	0.277	0.349	0.338
4	0.524	1.000	0.544	0.460	0.364	0.326	0.321	0.483
6	0.391	0.544	1.000	0.461	0.412	0.268	0.294	0.400
7	0.299	0.460	0.461	1.000	0.417	0.323	0.239	0.422
8	0.369	0.364	0.412	0.417	1.000	0.320	0.335	0.305
9	0.277	0.326	0.268	0.323	0.320	1.000	0.174	0.311
12	0.349	0.321	0.294	0.239	0.335	0.174	1.000	0.216
13	0.338	0.483	0.400	0.422	0.305	0.311	0.216	1.000

Spearman's rank correlation coefficient between items in the social cohesion subscale, calculated using pairwise complete observations.

### Ecometrics

Model building begins by investigating the sources of variability present in the data. It has already been observed that there is considerable variability among the question items (Figure 1). By fitting a generalised linear model to each ED, we discovered that at the contextual level of the model, too, there was evidence of variation; that is, there were differences observed between EDs as well as within EDs.

Within EDs, we plotted the responses of individuals, and there were some encouraging commonalities. For instance, some groups tended not to describe their neighbourhood in the most positive way possible, while others were less hesitant. Despite sharing such features, there was still substantial variability in individual responses, and any model should allow for this.

The picture presented by these exploratory analyses was one of considerable heterogeneity of responses at each of the question, individual and area levels. Graphical procedures are useful for teasing out this structure in the data; to quantify these sources of variability a formal model is required. For each neighbourhood cohesion subscale, a null (covariate-free) submodel of (1) was fitted using R via the Laplacian quadrature approximation to the likelihood. Table 5 shows the estimated parameters from these null models. In both this and the subsequent table, neighbourhood belonging and social cohesion models are treated entirely separately and do not share any parameters. In the models which included covariates, we found that omitting individuals with missing income information resulted in similar parameter estimates while giving increased convergence stability and better model fits. Further graphical explorations suggested that all models were a good fit to the data.

**Table 5:** Estimated parameters in covariate-free multilevel models

Parameter	Description	Neighbourhood Belonging	Social Cohesion
θ^ MathType@MTEF@5@5@+=feaafiart1ev1aaatCvAUfKttLearuWrP9MDH5MBPbIqV92AaeXatLxBI9gBaebbnrfifHhDYfgasaacH8akY=wiFfYdH8Gipec8Eeeu0xXdbba9frFj0=OqFfea0dXdd9vqai=hGuQ8kuc9pgc9s8qqaq=dirpe0xb9q8qiLsFr0=vr0=vr0dc8meaabaqaciaacaGaaeqabaqabeGadaaakeaaiiGacuWF4oqCgaqcaaaa@2E79@_1_	baseline parameter 1	-4.890 (-4.985, -4.795)	-3.698 (-3.744, -3.652)
θ^ MathType@MTEF@5@5@+=feaafiart1ev1aaatCvAUfKttLearuWrP9MDH5MBPbIqV92AaeXatLxBI9gBaebbnrfifHhDYfgasaacH8akY=wiFfYdH8Gipec8Eeeu0xXdbba9frFj0=OqFfea0dXdd9vqai=hGuQ8kuc9pgc9s8qqaq=dirpe0xb9q8qiLsFr0=vr0=vr0dc8meaabaqaciaacaGaaeqabaqabeGadaaakeaaiiGacuWF4oqCgaqcaaaa@2E79@_2_	baseline parameter 2	-3.718 (-3.808, -3.628)	-2.574 (-2.614, -2.535)
θ^ MathType@MTEF@5@5@+=feaafiart1ev1aaatCvAUfKttLearuWrP9MDH5MBPbIqV92AaeXatLxBI9gBaebbnrfifHhDYfgasaacH8akY=wiFfYdH8Gipec8Eeeu0xXdbba9frFj0=OqFfea0dXdd9vqai=hGuQ8kuc9pgc9s8qqaq=dirpe0xb9q8qiLsFr0=vr0=vr0dc8meaabaqaciaacaGaaeqabaqabeGadaaakeaaiiGacuWF4oqCgaqcaaaa@2E79@_3_	baseline parameter 3	-1.368 (-1.453, -1.282)	-1.430 (-1.468, -1.393)
θ^ MathType@MTEF@5@5@+=feaafiart1ev1aaatCvAUfKttLearuWrP9MDH5MBPbIqV92AaeXatLxBI9gBaebbnrfifHhDYfgasaacH8akY=wiFfYdH8Gipec8Eeeu0xXdbba9frFj0=OqFfea0dXdd9vqai=hGuQ8kuc9pgc9s8qqaq=dirpe0xb9q8qiLsFr0=vr0=vr0dc8meaabaqaciaacaGaaeqabaqabeGadaaakeaaiiGacuWF4oqCgaqcaaaa@2E79@_4_	baseline parameter 4	1.548 (1.462, 1.634)	1.338 (1.300, 1.376)
			
γ^ MathType@MTEF@5@5@+=feaafiart1ev1aaatCvAUfKttLearuWrP9MDH5MBPbIqV92AaeXatLxBI9gBaebbnrfifHhDYfgasaacH8akY=wiFfYdH8Gipec8Eeeu0xXdbba9frFj0=OqFfea0dXdd9vqai=hGuQ8kuc9pgc9s8qqaq=dirpe0xb9q8qiLsFr0=vr0=vr0dc8meaabaqaciaacaGaaeqabaqabeGadaaakeaaiiGacuWFZoWzgaqcaaaa@2E6A@_1_	attracted to n'hood	-0.201 (-0.232, -0.170)	
γ^ MathType@MTEF@5@5@+=feaafiart1ev1aaatCvAUfKttLearuWrP9MDH5MBPbIqV92AaeXatLxBI9gBaebbnrfifHhDYfgasaacH8akY=wiFfYdH8Gipec8Eeeu0xXdbba9frFj0=OqFfea0dXdd9vqai=hGuQ8kuc9pgc9s8qqaq=dirpe0xb9q8qiLsFr0=vr0=vr0dc8meaabaqaciaacaGaaeqabaqabeGadaaakeaaiiGacuWFZoWzgaqcaaaa@2E6A@_2_	belong to n'hood	-0.131 (-0.162, -0.100)	
γ^ MathType@MTEF@5@5@+=feaafiart1ev1aaatCvAUfKttLearuWrP9MDH5MBPbIqV92AaeXatLxBI9gBaebbnrfifHhDYfgasaacH8akY=wiFfYdH8Gipec8Eeeu0xXdbba9frFj0=OqFfea0dXdd9vqai=hGuQ8kuc9pgc9s8qqaq=dirpe0xb9q8qiLsFr0=vr0=vr0dc8meaabaqaciaacaGaaeqabaqabeGadaaakeaaiiGacuWFZoWzgaqcaaaa@2E6A@_3_	visit friends		0.120 (0.091, 0.148)
γ^ MathType@MTEF@5@5@+=feaafiart1ev1aaatCvAUfKttLearuWrP9MDH5MBPbIqV92AaeXatLxBI9gBaebbnrfifHhDYfgasaacH8akY=wiFfYdH8Gipec8Eeeu0xXdbba9frFj0=OqFfea0dXdd9vqai=hGuQ8kuc9pgc9s8qqaq=dirpe0xb9q8qiLsFr0=vr0=vr0dc8meaabaqaciaacaGaaeqabaqabeGadaaakeaaiiGacuWFZoWzgaqcaaaa@2E6A@_4_	friendships mean a lot		-0.235 (-0.264, -0.206)
γ^ MathType@MTEF@5@5@+=feaafiart1ev1aaatCvAUfKttLearuWrP9MDH5MBPbIqV92AaeXatLxBI9gBaebbnrfifHhDYfgasaacH8akY=wiFfYdH8Gipec8Eeeu0xXdbba9frFj0=OqFfea0dXdd9vqai=hGuQ8kuc9pgc9s8qqaq=dirpe0xb9q8qiLsFr0=vr0=vr0dc8meaabaqaciaacaGaaeqabaqabeGadaaakeaaiiGacuWFZoWzgaqcaaaa@2E6A@_5_	would like to move	0.428 (0.396, 0.459)	
γ^ MathType@MTEF@5@5@+=feaafiart1ev1aaatCvAUfKttLearuWrP9MDH5MBPbIqV92AaeXatLxBI9gBaebbnrfifHhDYfgasaacH8akY=wiFfYdH8Gipec8Eeeu0xXdbba9frFj0=OqFfea0dXdd9vqai=hGuQ8kuc9pgc9s8qqaq=dirpe0xb9q8qiLsFr0=vr0=vr0dc8meaabaqaciaacaGaaeqabaqabeGadaaakeaaiiGacuWFZoWzgaqcaaaa@2E6A@_6_	could go for advice		0.422 (0.394, 0.450)
γ^ MathType@MTEF@5@5@+=feaafiart1ev1aaatCvAUfKttLearuWrP9MDH5MBPbIqV92AaeXatLxBI9gBaebbnrfifHhDYfgasaacH8akY=wiFfYdH8Gipec8Eeeu0xXdbba9frFj0=OqFfea0dXdd9vqai=hGuQ8kuc9pgc9s8qqaq=dirpe0xb9q8qiLsFr0=vr0=vr0dc8meaabaqaciaacaGaaeqabaqabeGadaaakeaaiiGacuWFZoWzgaqcaaaa@2E6A@_7_	n'bours help in emergency		-1.379 (-1.411, -1.346)
γ^ MathType@MTEF@5@5@+=feaafiart1ev1aaatCvAUfKttLearuWrP9MDH5MBPbIqV92AaeXatLxBI9gBaebbnrfifHhDYfgasaacH8akY=wiFfYdH8Gipec8Eeeu0xXdbba9frFj0=OqFfea0dXdd9vqai=hGuQ8kuc9pgc9s8qqaq=dirpe0xb9q8qiLsFr0=vr0=vr0dc8meaabaqaciaacaGaaeqabaqabeGadaaakeaaiiGacuWFZoWzgaqcaaaa@2E6A@_8_	exchange favours with n'bours		0.801 (0.773, 0.830)
γ^ MathType@MTEF@5@5@+=feaafiart1ev1aaatCvAUfKttLearuWrP9MDH5MBPbIqV92AaeXatLxBI9gBaebbnrfifHhDYfgasaacH8akY=wiFfYdH8Gipec8Eeeu0xXdbba9frFj0=OqFfea0dXdd9vqai=hGuQ8kuc9pgc9s8qqaq=dirpe0xb9q8qiLsFr0=vr0=vr0dc8meaabaqaciaacaGaaeqabaqabeGadaaakeaaiiGacuWFZoWzgaqcaaaa@2E6A@_9_	work together to improve		-0.256 (-0.285, -0.227)
γ^ MathType@MTEF@5@5@+=feaafiart1ev1aaatCvAUfKttLearuWrP9MDH5MBPbIqV92AaeXatLxBI9gBaebbnrfifHhDYfgasaacH8akY=wiFfYdH8Gipec8Eeeu0xXdbba9frFj0=OqFfea0dXdd9vqai=hGuQ8kuc9pgc9s8qqaq=dirpe0xb9q8qiLsFr0=vr0=vr0dc8meaabaqaciaacaGaaeqabaqabeGadaaakeaaiiGacuWFZoWzgaqcaaaa@2E6A@_10_	plan to remain resident	-0.546 (-0.578, -0.514)	
γ^ MathType@MTEF@5@5@+=feaafiart1ev1aaatCvAUfKttLearuWrP9MDH5MBPbIqV92AaeXatLxBI9gBaebbnrfifHhDYfgasaacH8akY=wiFfYdH8Gipec8Eeeu0xXdbba9frFj0=OqFfea0dXdd9vqai=hGuQ8kuc9pgc9s8qqaq=dirpe0xb9q8qiLsFr0=vr0=vr0dc8meaabaqaciaacaGaaeqabaqabeGadaaakeaaiiGacuWFZoWzgaqcaaaa@2E6A@_11_	similar to people in n'hood	-0.229 (-0.260, -0.198)	
γ^ MathType@MTEF@5@5@+=feaafiart1ev1aaatCvAUfKttLearuWrP9MDH5MBPbIqV92AaeXatLxBI9gBaebbnrfifHhDYfgasaacH8akY=wiFfYdH8Gipec8Eeeu0xXdbba9frFj0=OqFfea0dXdd9vqai=hGuQ8kuc9pgc9s8qqaq=dirpe0xb9q8qiLsFr0=vr0=vr0dc8meaabaqaciaacaGaaeqabaqabeGadaaakeaaiiGacuWFZoWzgaqcaaaa@2E6A@_12_	rarely have n'bour visit		1.160 (1.132, 1.189)
γ^ MathType@MTEF@5@5@+=feaafiart1ev1aaatCvAUfKttLearuWrP9MDH5MBPbIqV92AaeXatLxBI9gBaebbnrfifHhDYfgasaacH8akY=wiFfYdH8Gipec8Eeeu0xXdbba9frFj0=OqFfea0dXdd9vqai=hGuQ8kuc9pgc9s8qqaq=dirpe0xb9q8qiLsFr0=vr0=vr0dc8meaabaqaciaacaGaaeqabaqabeGadaaakeaaiiGacuWFZoWzgaqcaaaa@2E6A@_13_	regularly talk with people		-0.633 (-0.663, -0.603)
γ^ MathType@MTEF@5@5@+=feaafiart1ev1aaatCvAUfKttLearuWrP9MDH5MBPbIqV92AaeXatLxBI9gBaebbnrfifHhDYfgasaacH8akY=wiFfYdH8Gipec8Eeeu0xXdbba9frFj0=OqFfea0dXdd9vqai=hGuQ8kuc9pgc9s8qqaq=dirpe0xb9q8qiLsFr0=vr0=vr0dc8meaabaqaciaacaGaaeqabaqabeGadaaakeaaiiGacuWFZoWzgaqcaaaa@2E6A@_14_	sense of community	0.402 (0.371, 0.432)	
γ^ MathType@MTEF@5@5@+=feaafiart1ev1aaatCvAUfKttLearuWrP9MDH5MBPbIqV92AaeXatLxBI9gBaebbnrfifHhDYfgasaacH8akY=wiFfYdH8Gipec8Eeeu0xXdbba9frFj0=OqFfea0dXdd9vqai=hGuQ8kuc9pgc9s8qqaq=dirpe0xb9q8qiLsFr0=vr0=vr0dc8meaabaqaciaacaGaaeqabaqabeGadaaakeaaiiGacuWFZoWzgaqcaaaa@2E6A@_15_	good place for children	0.278 (0.247, 0.308)	
			
σ^U2 MathType@MTEF@5@5@+=feaafiart1ev1aaatCvAUfKttLearuWrP9MDH5MBPbIqV92AaeXatLxBI9gBaebbnrfifHhDYfgasaacH8akY=wiFfYdH8Gipec8Eeeu0xXdbba9frFj0=OqFfea0dXdd9vqai=hGuQ8kuc9pgc9s8qqaq=dirpe0xb9q8qiLsFr0=vr0=vr0dc8meaabaqaciaacaGaaeqabaqabeGadaaakeaaiiGacuWFdpWCgaqcamaaDaaaleaacqWGvbqvaeaacqaIYaGmaaaaaa@30D8@	ED-level variance	0.399 (0.355, 0.447)	0.029 (0.028, 0.031)
σ^V2 MathType@MTEF@5@5@+=feaafiart1ev1aaatCvAUfKttLearuWrP9MDH5MBPbIqV92AaeXatLxBI9gBaebbnrfifHhDYfgasaacH8akY=wiFfYdH8Gipec8Eeeu0xXdbba9frFj0=OqFfea0dXdd9vqai=hGuQ8kuc9pgc9s8qqaq=dirpe0xb9q8qiLsFr0=vr0=vr0dc8meaabaqaciaacaGaaeqabaqabeGadaaakeaaiiGacuWFdpWCgaqcamaaDaaaleaacqWGwbGvaeaacqaIYaGmaaaaaa@30DA@	individual-level variance	4.773 (4.663, 4.886)	1.829 (1.785, 1.871)

Estimates of fixed-effect coefficients are on the log-odds scale. 95% confidence intervals are given in parentheses. The abbreviations n'hood, n'bour(s) and ED refer, respectively, to neighbourhood, neighbour(s) and enumeration district.

The first thing that will be noted is the apparent reversal of the ideals of ecometric analysis [17]: for both subscales the largest amount of variability is to be found at the individual level (σ^V
 MathType@MTEF@5@5@+=feaafiart1ev1aaatCvAUfKttLearuWrP9MDH5MBPbIqV92AaeXatLxBI9gBaebbnrfifHhDYfgasaacH8akY=wiFfYdH8Gipec8Eeeu0xXdbba9frFj0=OqFfea0dXdd9vqai=hGuQ8kuc9pgc9s8qqaq=dirpe0xb9q8qiLsFr0=vr0=vr0dc8meaabaqaciaacaGaaeqabaqabeGadaaakeaaiiGacuWFdpWCgaqcamaaBaaaleaacqWGwbGvaeqaaaaa@2FE7@). This phenomenon is not new, however; Wainwright and Surtees [38] note that "the extent of area level relative to individual level variation is usually modest" and even Raudenbush and Sampson [17] acknowledge that "it is clear that in no case does most of the variation ... lie between neighbourhoods". Pickett and Pearl [39] and Merlo [40] echo this observation, for which there are a number of possible explanations. A behavioural line of reasoning suggests that it is individuals, not neighbourhoods, who are neighbourly and who feel they belong in an area. If so, individuals could be considered to be sampled at random from the entire population, rather than from within regions. From an ecometric perspective, a complementary observation is that in answering a questionnaire, each individual is both the assessor and the assessed: they are required to *interpret *and then *compare *themselves against each five-point scale. There is, therefore, a hidden layer of heterogeneity absorbed into the individual-level variability, explaining some of its magnitude.

The estimated fixed effects θ^
 MathType@MTEF@5@5@+=feaafiart1ev1aaatCvAUfKttLearuWrP9MDH5MBPbIqV92AaeXatLxBI9gBaebbnrfifHhDYfgasaacH8akY=wiFfYdH8Gipec8Eeeu0xXdbba9frFj0=OqFfea0dXdd9vqai=hGuQ8kuc9pgc9s8qqaq=dirpe0xb9q8qiLsFr0=vr0=vr0dc8meaabaqaciaacaGaaeqabaqabeGadaaakeaaiiGacuWF4oqCgaqcaaaa@2E79@_1_,...,θ^
 MathType@MTEF@5@5@+=feaafiart1ev1aaatCvAUfKttLearuWrP9MDH5MBPbIqV92AaeXatLxBI9gBaebbnrfifHhDYfgasaacH8akY=wiFfYdH8Gipec8Eeeu0xXdbba9frFj0=OqFfea0dXdd9vqai=hGuQ8kuc9pgc9s8qqaq=dirpe0xb9q8qiLsFr0=vr0=vr0dc8meaabaqaciaacaGaaeqabaqabeGadaaakeaaiiGacuWF4oqCgaqcaaaa@2E79@_4 _were consistent with the skew shown in Figure 1. The estimated scale of the model – that is, the ratio of the response variance to the nominal binomial variance – was very nearly equal to unity in both cases, suggesting that the binomial model captured dispersion adequately. To assess whether the variation at the three levels of the model could be explained by individual-level covariates, we added a number of variables found in univariate analyses to be associated with neighbourhood cohesion. In some senses, it would be preferable if these were unimportant in explaining the residual variation present in the item responses. In such cases neighbourhood cohesion, quantified by one or both subscales, could be used and thought of as an independent explanatory variable, albeit measured with error due to variability at the individual level. If, conversely, the inclusion of covariates resulted in significant reduction in unexplained variation, then inclusion of these covariates alongside neighbourhood cohesion in further models could result in correlated regression parameters and potential interpretative difficulties. Alternatively or additionally, including covariates in model (1) could lead to a reduction in the variation previously explained by the random effects. By the criteria of Raudenbush and Sampson [17], this is desirable at the individual level but undesirable at the area and item levels.

Table 6 gives parameter estimates from model (1) when covariates are included. Encouragingly, the item-and area-level random effects variances decreased by less than 25% from those in Table 5; on the other hand, neither are the individual-level variances diminished substantially. Unsurprisingly given the size of the dataset under consideration, many of the effects were statistically significant at the 5% level. Perhaps more important in such cases is determining which, if any, have a noticeable impact on the linear predictor *η*_*ijk*_. Most covariates had only small effects; notable exceptions included students, who were subtantially less likely to feel they belong to a neighbourhood. This effect may be negated somewhat by the positive belonging of the social class categorisation 'other', a group comprised mainly of individuals not in employment or who are economically inactive, and once again including students. Among the covariates associated with the social cohesion subscale, individuals who reported permanent sickness or disability showed substantially less cohesion than employees.

**Table 6:** Estimated parameters in multilevel models with covariates

Parameter	Description	Neighbourhood Belonging	Social Cohesion
θ^ MathType@MTEF@5@5@+=feaafiart1ev1aaatCvAUfKttLearuWrP9MDH5MBPbIqV92AaeXatLxBI9gBaebbnrfifHhDYfgasaacH8akY=wiFfYdH8Gipec8Eeeu0xXdbba9frFj0=OqFfea0dXdd9vqai=hGuQ8kuc9pgc9s8qqaq=dirpe0xb9q8qiLsFr0=vr0=vr0dc8meaabaqaciaacaGaaeqabaqabeGadaaakeaaiiGacuWF4oqCgaqcaaaa@2E79@_1_	baseline parameter 1	-4.926 (-5.173, -4.680)	-3.686 (-3.838, -3.533)
θ^ MathType@MTEF@5@5@+=feaafiart1ev1aaatCvAUfKttLearuWrP9MDH5MBPbIqV92AaeXatLxBI9gBaebbnrfifHhDYfgasaacH8akY=wiFfYdH8Gipec8Eeeu0xXdbba9frFj0=OqFfea0dXdd9vqai=hGuQ8kuc9pgc9s8qqaq=dirpe0xb9q8qiLsFr0=vr0=vr0dc8meaabaqaciaacaGaaeqabaqabeGadaaakeaaiiGacuWF4oqCgaqcaaaa@2E79@_2_	baseline parameter 2	-3.742 (-3.986, -3.497)	-2.552 (-2.703, -2.401)
θ^ MathType@MTEF@5@5@+=feaafiart1ev1aaatCvAUfKttLearuWrP9MDH5MBPbIqV92AaeXatLxBI9gBaebbnrfifHhDYfgasaacH8akY=wiFfYdH8Gipec8Eeeu0xXdbba9frFj0=OqFfea0dXdd9vqai=hGuQ8kuc9pgc9s8qqaq=dirpe0xb9q8qiLsFr0=vr0=vr0dc8meaabaqaciaacaGaaeqabaqabeGadaaakeaaiiGacuWF4oqCgaqcaaaa@2E79@_3_	baseline parameter 3	-1.369 (-1.612, -1.127)	-1.398 (-1.549, -1.248)
θ^ MathType@MTEF@5@5@+=feaafiart1ev1aaatCvAUfKttLearuWrP9MDH5MBPbIqV92AaeXatLxBI9gBaebbnrfifHhDYfgasaacH8akY=wiFfYdH8Gipec8Eeeu0xXdbba9frFj0=OqFfea0dXdd9vqai=hGuQ8kuc9pgc9s8qqaq=dirpe0xb9q8qiLsFr0=vr0=vr0dc8meaabaqaciaacaGaaeqabaqabeGadaaakeaaiiGacuWF4oqCgaqcaaaa@2E79@_4_	baseline parameter 4	1.526 (1.283, 1.769)	1.371 (1.221, 1.521)
			
γ^ MathType@MTEF@5@5@+=feaafiart1ev1aaatCvAUfKttLearuWrP9MDH5MBPbIqV92AaeXatLxBI9gBaebbnrfifHhDYfgasaacH8akY=wiFfYdH8Gipec8Eeeu0xXdbba9frFj0=OqFfea0dXdd9vqai=hGuQ8kuc9pgc9s8qqaq=dirpe0xb9q8qiLsFr0=vr0=vr0dc8meaabaqaciaacaGaaeqabaqabeGadaaakeaaiiGacuWFZoWzgaqcaaaa@2E6A@_1_	attracted to n'hood	-0.215 (-0.247, -0.183)	
γ^ MathType@MTEF@5@5@+=feaafiart1ev1aaatCvAUfKttLearuWrP9MDH5MBPbIqV92AaeXatLxBI9gBaebbnrfifHhDYfgasaacH8akY=wiFfYdH8Gipec8Eeeu0xXdbba9frFj0=OqFfea0dXdd9vqai=hGuQ8kuc9pgc9s8qqaq=dirpe0xb9q8qiLsFr0=vr0=vr0dc8meaabaqaciaacaGaaeqabaqabeGadaaakeaaiiGacuWFZoWzgaqcaaaa@2E6A@_2_	belong to n'hood	-0.123 (-0.155, -0.091)	
γ^ MathType@MTEF@5@5@+=feaafiart1ev1aaatCvAUfKttLearuWrP9MDH5MBPbIqV92AaeXatLxBI9gBaebbnrfifHhDYfgasaacH8akY=wiFfYdH8Gipec8Eeeu0xXdbba9frFj0=OqFfea0dXdd9vqai=hGuQ8kuc9pgc9s8qqaq=dirpe0xb9q8qiLsFr0=vr0=vr0dc8meaabaqaciaacaGaaeqabaqabeGadaaakeaaiiGacuWFZoWzgaqcaaaa@2E6A@_3_	visit friends		0.126 (0.096, 0.156)
γ^ MathType@MTEF@5@5@+=feaafiart1ev1aaatCvAUfKttLearuWrP9MDH5MBPbIqV92AaeXatLxBI9gBaebbnrfifHhDYfgasaacH8akY=wiFfYdH8Gipec8Eeeu0xXdbba9frFj0=OqFfea0dXdd9vqai=hGuQ8kuc9pgc9s8qqaq=dirpe0xb9q8qiLsFr0=vr0=vr0dc8meaabaqaciaacaGaaeqabaqabeGadaaakeaaiiGacuWFZoWzgaqcaaaa@2E6A@_4_	friendships mean a lot		-0.228 (-0.257, -0.198)
γ^ MathType@MTEF@5@5@+=feaafiart1ev1aaatCvAUfKttLearuWrP9MDH5MBPbIqV92AaeXatLxBI9gBaebbnrfifHhDYfgasaacH8akY=wiFfYdH8Gipec8Eeeu0xXdbba9frFj0=OqFfea0dXdd9vqai=hGuQ8kuc9pgc9s8qqaq=dirpe0xb9q8qiLsFr0=vr0=vr0dc8meaabaqaciaacaGaaeqabaqabeGadaaakeaaiiGacuWFZoWzgaqcaaaa@2E6A@_5_	would like to move	0.429 (0.396, 0.461)	
γ^ MathType@MTEF@5@5@+=feaafiart1ev1aaatCvAUfKttLearuWrP9MDH5MBPbIqV92AaeXatLxBI9gBaebbnrfifHhDYfgasaacH8akY=wiFfYdH8Gipec8Eeeu0xXdbba9frFj0=OqFfea0dXdd9vqai=hGuQ8kuc9pgc9s8qqaq=dirpe0xb9q8qiLsFr0=vr0=vr0dc8meaabaqaciaacaGaaeqabaqabeGadaaakeaaiiGacuWFZoWzgaqcaaaa@2E6A@_6_	could go for advice		0.437 (0.408, 0.466)
γ^ MathType@MTEF@5@5@+=feaafiart1ev1aaatCvAUfKttLearuWrP9MDH5MBPbIqV92AaeXatLxBI9gBaebbnrfifHhDYfgasaacH8akY=wiFfYdH8Gipec8Eeeu0xXdbba9frFj0=OqFfea0dXdd9vqai=hGuQ8kuc9pgc9s8qqaq=dirpe0xb9q8qiLsFr0=vr0=vr0dc8meaabaqaciaacaGaaeqabaqabeGadaaakeaaiiGacuWFZoWzgaqcaaaa@2E6A@_7_	n'bours help in emergency		-1.381 (-1.414, -1.347)
γ^ MathType@MTEF@5@5@+=feaafiart1ev1aaatCvAUfKttLearuWrP9MDH5MBPbIqV92AaeXatLxBI9gBaebbnrfifHhDYfgasaacH8akY=wiFfYdH8Gipec8Eeeu0xXdbba9frFj0=OqFfea0dXdd9vqai=hGuQ8kuc9pgc9s8qqaq=dirpe0xb9q8qiLsFr0=vr0=vr0dc8meaabaqaciaacaGaaeqabaqabeGadaaakeaaiiGacuWFZoWzgaqcaaaa@2E6A@_8_	exchange favours with n'bours		0.775 (0.745, 0.804)
γ^ MathType@MTEF@5@5@+=feaafiart1ev1aaatCvAUfKttLearuWrP9MDH5MBPbIqV92AaeXatLxBI9gBaebbnrfifHhDYfgasaacH8akY=wiFfYdH8Gipec8Eeeu0xXdbba9frFj0=OqFfea0dXdd9vqai=hGuQ8kuc9pgc9s8qqaq=dirpe0xb9q8qiLsFr0=vr0=vr0dc8meaabaqaciaacaGaaeqabaqabeGadaaakeaaiiGacuWFZoWzgaqcaaaa@2E6A@_9_	work together to improve		-0.273 (-0.303, -0.243)
γ^ MathType@MTEF@5@5@+=feaafiart1ev1aaatCvAUfKttLearuWrP9MDH5MBPbIqV92AaeXatLxBI9gBaebbnrfifHhDYfgasaacH8akY=wiFfYdH8Gipec8Eeeu0xXdbba9frFj0=OqFfea0dXdd9vqai=hGuQ8kuc9pgc9s8qqaq=dirpe0xb9q8qiLsFr0=vr0=vr0dc8meaabaqaciaacaGaaeqabaqabeGadaaakeaaiiGacuWFZoWzgaqcaaaa@2E6A@_10_	plan to remain resident	-0.547 (-0.580, -0.513)	
γ^ MathType@MTEF@5@5@+=feaafiart1ev1aaatCvAUfKttLearuWrP9MDH5MBPbIqV92AaeXatLxBI9gBaebbnrfifHhDYfgasaacH8akY=wiFfYdH8Gipec8Eeeu0xXdbba9frFj0=OqFfea0dXdd9vqai=hGuQ8kuc9pgc9s8qqaq=dirpe0xb9q8qiLsFr0=vr0=vr0dc8meaabaqaciaacaGaaeqabaqabeGadaaakeaaiiGacuWFZoWzgaqcaaaa@2E6A@_11_	similar to people in n'hood	-0.231 (-0.263, -0.198)	
γ^ MathType@MTEF@5@5@+=feaafiart1ev1aaatCvAUfKttLearuWrP9MDH5MBPbIqV92AaeXatLxBI9gBaebbnrfifHhDYfgasaacH8akY=wiFfYdH8Gipec8Eeeu0xXdbba9frFj0=OqFfea0dXdd9vqai=hGuQ8kuc9pgc9s8qqaq=dirpe0xb9q8qiLsFr0=vr0=vr0dc8meaabaqaciaacaGaaeqabaqabeGadaaakeaaiiGacuWFZoWzgaqcaaaa@2E6A@_12_	rarely have n'bour visit		1.168 (1.139, 1.198)
γ^ MathType@MTEF@5@5@+=feaafiart1ev1aaatCvAUfKttLearuWrP9MDH5MBPbIqV92AaeXatLxBI9gBaebbnrfifHhDYfgasaacH8akY=wiFfYdH8Gipec8Eeeu0xXdbba9frFj0=OqFfea0dXdd9vqai=hGuQ8kuc9pgc9s8qqaq=dirpe0xb9q8qiLsFr0=vr0=vr0dc8meaabaqaciaacaGaaeqabaqabeGadaaakeaaiiGacuWFZoWzgaqcaaaa@2E6A@_13_	regularly talk with people		-0.625 (-0.656, -0.594)
γ^ MathType@MTEF@5@5@+=feaafiart1ev1aaatCvAUfKttLearuWrP9MDH5MBPbIqV92AaeXatLxBI9gBaebbnrfifHhDYfgasaacH8akY=wiFfYdH8Gipec8Eeeu0xXdbba9frFj0=OqFfea0dXdd9vqai=hGuQ8kuc9pgc9s8qqaq=dirpe0xb9q8qiLsFr0=vr0=vr0dc8meaabaqaciaacaGaaeqabaqabeGadaaakeaaiiGacuWFZoWzgaqcaaaa@2E6A@_14_	sense of community	0.416 (0.384, 0.447)	
γ^ MathType@MTEF@5@5@+=feaafiart1ev1aaatCvAUfKttLearuWrP9MDH5MBPbIqV92AaeXatLxBI9gBaebbnrfifHhDYfgasaacH8akY=wiFfYdH8Gipec8Eeeu0xXdbba9frFj0=OqFfea0dXdd9vqai=hGuQ8kuc9pgc9s8qqaq=dirpe0xb9q8qiLsFr0=vr0=vr0dc8meaabaqaciaacaGaaeqabaqabeGadaaakeaaiiGacuWFZoWzgaqcaaaa@2E6A@_15_	good place for children	0.271 (0.239, 0.303)	
			
β^ MathType@MTEF@5@5@+=feaafiart1ev1aaatCvAUfKttLearuWrP9MDH5MBPbIqV92AaeXatLxBI9gBaebbnrfifHhDYfgasaacH8akY=wiFfYdH8Gipec8Eeeu0xXdbba9frFj0=OqFfea0dXdd9vqai=hGuQ8kuc9pgc9s8qqaq=dirpe0xb9q8qiLsFr0=vr0=vr0dc8meaabaqaciaacaGaaeqabaqabeGadaaakeaaiiGacuWFYoGygaqcaaaa@2E64@	centred age	0.028 (0.024, 0.032)	0.005 (0.003, 0.008)
	male	-0.211 (-0.307, -0.115)	-0.131 (-0.193, -0.069)
	social class: IIINM	0.031 (-0.109, 0.171)	-0.023 (-0.113, 0.066)
	social class: IIIM	0.360 (0.218, 0.501)	0.090 (-0.001, 0.181)
	social class: IV&V	0.282 (0.145, 0.419)	0.079 (-0.009, 0.167)
	social class: other	0.463 (0.236, 0.690)	0.052 (-0.094, 0.198)
	social class: missing	0.280 (0.079, 0.482)	0.040 (-0.090, 0.170)
	council tax: A&B	-0.367 (-0.489, -0.245)	-0.041 (-0.112, 0.030)
	council tax: missing	-0.302 (-0.469, -0.135)	-0.083 (-0.187, 0.020)
	employment status: seeking	-0.339 (-0.634, -0.044)	-0.101 (-0.292, 0.090)
	employment status: student	-0.737 (-1.099, -0.375)	-0.037 (-0.271, 0.197)
	employment status: home/carer	-0.076 (-0.267, 0.115)	-0.001 (-0.124, 0.122)
	employment status: disability	-0.041 (-0.208, 0.125)	-0.235 (-0.342, -0.128)
	employment status: retired	0.086 (-0.072, 0.245)	0.056 (-0.046, 0.158)
	employment status: missing	0.285 (0.053, 0.517)	0.109 (-0.041, 0.259)
	gross income: >£95, <£215/week	-0.081 (-0.234, 0.072)	-0.040 (-0.139, 0.059)
	gross income: >£215/week	-0.098 (-0.272, 0.076)	-0.007 (-0.119, 0.106)
	tenancy: owner	0.280 (0.153, 0.407)	0.156 (0.076, 0.237)
	tenancy: missing	0.184 (-0.209, 0.577)	0.014 (-0.238, 0.266)
			
σ^U2 MathType@MTEF@5@5@+=feaafiart1ev1aaatCvAUfKttLearuWrP9MDH5MBPbIqV92AaeXatLxBI9gBaebbnrfifHhDYfgasaacH8akY=wiFfYdH8Gipec8Eeeu0xXdbba9frFj0=OqFfea0dXdd9vqai=hGuQ8kuc9pgc9s8qqaq=dirpe0xb9q8qiLsFr0=vr0=vr0dc8meaabaqaciaacaGaaeqabaqabeGadaaakeaaiiGacuWFdpWCgaqcamaaDaaaleaacqWGvbqvaeaacqaIYaGmaaaaaa@30D8@	ED-level variance	0.307 (0.276, 0.339)	0.019 (0.018, 0.019)
σ^V2 MathType@MTEF@5@5@+=feaafiart1ev1aaatCvAUfKttLearuWrP9MDH5MBPbIqV92AaeXatLxBI9gBaebbnrfifHhDYfgasaacH8akY=wiFfYdH8Gipec8Eeeu0xXdbba9frFj0=OqFfea0dXdd9vqai=hGuQ8kuc9pgc9s8qqaq=dirpe0xb9q8qiLsFr0=vr0=vr0dc8meaabaqaciaacaGaaeqabaqabeGadaaakeaaiiGacuWFdpWCgaqcamaaDaaaleaacqWGwbGvaeaacqaIYaGmaaaaaa@30DA@	individual-level variance	4.503 (4.396, 4.604)	1.822 (1.778, 1.868)

Reference categories are females, social classes I & II, council tax bands C-H, employed persons, with gross household income less than £95 per week, and non-houseowners. Abbreviations are as in Table 5.

**Figure 3:**
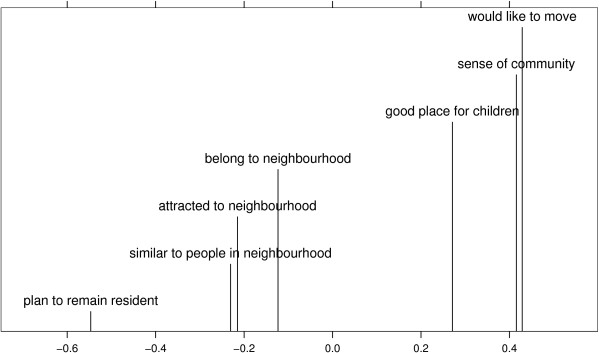
**Posterior estimated neighbourhood belonging random effects, at the item-level**. Estimates are on the log-odds scale, with large positive values corresponding to distinctive behaviour patterns. A score of 0.1, for example, corresponds to the odds of not continuing to a higher category, as opposed to continuing, being increased by a factor of exp(0.1) ≈ 1.11, or around 11%.

**Figure 4:**
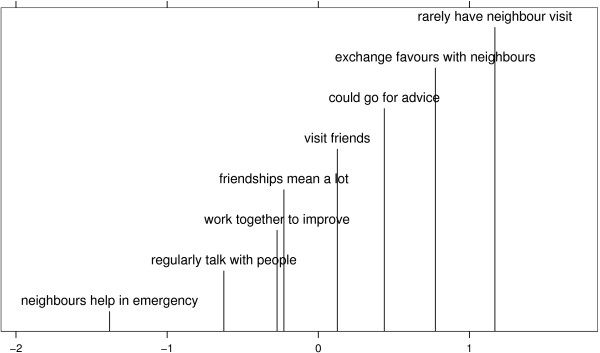
**Posterior estimated social cohesion random effects, at the item-level**. Estimates are on the log-odds scale, with large positive values corresponding to distinctive behaviour patterns. A score of 0.1, for example, corresponds to the odds of not continuing to a higher category, as opposed to continuing, being increased by a factor of exp(0.1) ≈ 1.11, or around 11%.

Consider now each level of variability in turn. Estimated item-level deviations are illustrated in Figure 3 and Figure 4, plots which should be examined with reference to Figure 1 and Table 1. The item-level deviations may be interpreted as quantifying the distinctiveness of the various activities associated with the scale items. Uncommon activities will appear at the right-hand end of the figure since, under the model defined by (1) and (2), they increase the chance of not progressing to a higher score on the five-point scale. Conversely, near-universal activities will be found at the left-hand end of the figure, since they correspondingly decrease the chance of not continuing to a higher scale score. In between, Raudenbush and Sampson [17] suggest that there should be an even spread of scale items, so that some are quite common, some neither common nor uncommon, some quite uncommon, and so on. Overall, there is more variability in the social cohesion items than in those forming the neighbourhood belonging subscale.

Figure 3 presents the counterintuitive idea that while many individuals plan to remain resident in their neighbourhood, it is also not uncommon to express a desire to move. Neighbourhood belonging itself is the median estimated random effect, a desirable result on this subscale. Broadly, there is a good spread of item-level deviations, with perhaps an undesirably large gap around the average neighbourhood belonging of zero.

In evidence once again in Figure 4 is the expression that neighbours will help in an emergency; at the other extreme it is common, apparently, to rarely have a neighbour come to visit. The social cohesion scale showed many of the desirable properties highlighted by Raudenbush and Sampson [17]: the items are evenly and widely spaced and fine discrimination is, in theory, possible using a scale derived from such items. Here the defining social cohesion item, borrowing and exchanging with neighbours, is near the upper end of the scale, indicating that this represents highly distinctive behaviour.

The reliability in estimation of the latent neighbourhood capitals *U*_1_,..., *U*_*I *_may be assessed by way of quantities closely related to the intra-neighbourhood correlations (INCs). Since the reliability of an estimator *Û*_*i *_of *U*_*i *_is defined [41] as

λi=Var(Ui)Var(U^i)     (4)
 MathType@MTEF@5@5@+=feaafiart1ev1aaatCvAUfKttLearuWrP9MDH5MBPbIqV92AaeXatLxBI9gBaebbnrfifHhDYfgasaacH8akY=wiFfYdH8Gipec8Eeeu0xXdbba9frFj0=OqFfea0dXdd9vqai=hGuQ8kuc9pgc9s8qqaq=dirpe0xb9q8qiLsFr0=vr0=vr0dc8meaabaqaciaacaGaaeqabaqabeGadaaakeaaiiGacqWF7oaBdaWgaaWcbaGaemyAaKgabeaakiabg2da9maalaaabaGaeeOvayLaeeyyaeMaeeOCaiNaeiikaGIaemyvau1aaSbaaSqaaiabdMgaPbqabaGccqGGPaqkaeaacqqGwbGvcqqGHbqycqqGYbGCcqGGOaakcuWGvbqvgaqcamaaBaaaleaacqWGPbqAaeqaaOGaeiykaKcaaiaaxMaacaWLjaWaaeWaaeaacqaI0aanaiaawIcacaGLPaaaaaa@459B@

setting U^i=Ui+Ji−1∑j=1JiVij
 MathType@MTEF@5@5@+=feaafiart1ev1aaatCvAUfKttLearuWrP9MDH5MBPbIqV92AaeXatLxBI9gBaebbnrfifHhDYfgasaacH8akY=wiFfYdH8Gipec8Eeeu0xXdbba9frFj0=OqFfea0dXdd9vqai=hGuQ8kuc9pgc9s8qqaq=dirpe0xb9q8qiLsFr0=vr0=vr0dc8meaabaqaciaacaGaaeqabaqabeGadaaakeaacuWGvbqvgaqcamaaBaaaleaacqWGPbqAaeqaaOGaeyypa0Jaemyvau1aaSbaaSqaaiabdMgaPbqabaGccqGHRaWkcqWGkbGsdaqhaaWcbaGaemyAaKgabaGaeyOeI0IaeGymaedaaOWaaabmaeaacqWGwbGvdaWgaaWcbaGaemyAaKMaemOAaOgabeaaaeaacqWGQbGAcqGH9aqpcqaIXaqmaeaacqWGkbGsdaWgaaadbaGaemyAaKgabeaaa0GaeyyeIuoaaaa@44C0@ (say) makes (4) equivalent to

λi=σU2σU2+σV2Ji.     (5)
 MathType@MTEF@5@5@+=feaafiart1ev1aaatCvAUfKttLearuWrP9MDH5MBPbIqV92AaeXatLxBI9gBaebbnrfifHhDYfgasaacH8akY=wiFfYdH8Gipec8Eeeu0xXdbba9frFj0=OqFfea0dXdd9vqai=hGuQ8kuc9pgc9s8qqaq=dirpe0xb9q8qiLsFr0=vr0=vr0dc8meaabaqaciaacaGaaeqabaqabeGadaaakeaaiiGacqWF7oaBdaWgaaWcbaGaemyAaKgabeaakiabg2da9maalaaabaGae83Wdm3aa0baaSqaaiabdwfavbqaaiabikdaYaaaaOqaaiab=n8aZnaaDaaaleaacqWGvbqvaeaacqaIYaGmaaGccqGHRaWkdaWcaaqaaiab=n8aZnaaDaaaleaacqWGwbGvaeaacqaIYaGmaaaakeaacqWGkbGsdaWgaaWcbaGaemyAaKgabeaaaaaaaOGaeiOla4IaaCzcaiaaxMaadaqadaqaaiabiwda1aGaayjkaiaawMcaaaaa@45A7@

Recall that σU2
 MathType@MTEF@5@5@+=feaafiart1ev1aaatCvAUfKttLearuWrP9MDH5MBPbIqV92AaeXatLxBI9gBaebbnrfifHhDYfgasaacH8akY=wiFfYdH8Gipec8Eeeu0xXdbba9frFj0=OqFfea0dXdd9vqai=hGuQ8kuc9pgc9s8qqaq=dirpe0xb9q8qiLsFr0=vr0=vr0dc8meaabaqaciaacaGaaeqabaqabeGadaaakeaaiiGacqWFdpWCdaqhaaWcbaGaemyvaufabaGaeGOmaidaaaaa@30C8@ and σV2
 MathType@MTEF@5@5@+=feaafiart1ev1aaatCvAUfKttLearuWrP9MDH5MBPbIqV92AaeXatLxBI9gBaebbnrfifHhDYfgasaacH8akY=wiFfYdH8Gipec8Eeeu0xXdbba9frFj0=OqFfea0dXdd9vqai=hGuQ8kuc9pgc9s8qqaq=dirpe0xb9q8qiLsFr0=vr0=vr0dc8meaabaqaciaacaGaaeqabaqabeGadaaakeaaiiGacqWFdpWCdaqhaaWcbaGaemOvayfabaGaeGOmaidaaaaa@30CA@ are the between-area and between-individual-within-area variances, respectively. The reliability (5) is similar in structure to the INC

σU2σU2+σV2.     (6)
 MathType@MTEF@5@5@+=feaafiart1ev1aaatCvAUfKttLearuWrP9MDH5MBPbIqV92AaeXatLxBI9gBaebbnrfifHhDYfgasaacH8akY=wiFfYdH8Gipec8Eeeu0xXdbba9frFj0=OqFfea0dXdd9vqai=hGuQ8kuc9pgc9s8qqaq=dirpe0xb9q8qiLsFr0=vr0=vr0dc8meaabaqaciaacaGaaeqabaqabeGadaaakeaadaWcaaqaaGGaciab=n8aZnaaDaaaleaacqWGvbqvaeaacqaIYaGmaaaakeaacqWFdpWCdaqhaaWcbaGaemyvaufabaGaeGOmaidaaOGaey4kaSIae83Wdm3aa0baaSqaaiabdAfawbqaaiabikdaYaaaaaGccqGGUaGlcaWLjaGaaCzcamaabmaabaGaeGOnaydacaGLOaGaayzkaaaaaa@3EA5@

Of course, the estimators *Û*_*i *_are idealised and cannot be computed in practice; nevertheless, they are very informative as to whether measurements on individuals provide reasonable estimates of neighbourhood capital. The reliability (5) may be thought of as an upper bound for the reliability of any estimator based on these data. Further, this generic reliability is likely to be more interpretable than estimator specific reliabilities, which may be entirely spurious: a constant estimator *Û*_*i*_*= c *has zero variance and thus infinite reliability, though (4) is clearly intended to be bounded between zero and one. The INC (6) may be estimated by

**Figure 5:**
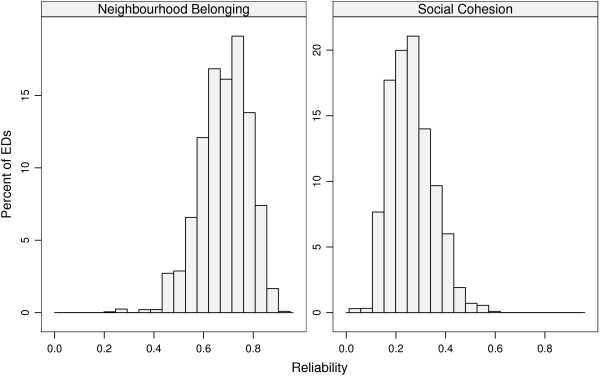
**Estimated reliability for Neighbourhood Belonging and Social Cohesion subscales**. Histograms of the reliabilities for the two neighbourhood cohesion subscales, accounting for uncertainty in both the number of individuals in an ED and the variability in the estimates of the variance parameters.

σ^U2σ^U2+σ^V2,     (7)
 MathType@MTEF@5@5@+=feaafiart1ev1aaatCvAUfKttLearuWrP9MDH5MBPbIqV92AaeXatLxBI9gBaebbnrfifHhDYfgasaacH8akY=wiFfYdH8Gipec8Eeeu0xXdbba9frFj0=OqFfea0dXdd9vqai=hGuQ8kuc9pgc9s8qqaq=dirpe0xb9q8qiLsFr0=vr0=vr0dc8meaabaqaciaacaGaaeqabaqabeGadaaakeaadaWcaaqaaGGaciqb=n8aZzaajaWaa0baaSqaaiabdwfavbqaaiabikdaYaaaaOqaaiqb=n8aZzaajaWaa0baaSqaaiabdwfavbqaaiabikdaYaaakiabgUcaRiqb=n8aZzaajaWaa0baaSqaaiabdAfawbqaaiabikdaYaaaaaGccqGGSaalcaWLjaGaaCzcamaabmaabaGaeG4naCdacaGLOaGaayzkaaaaaa@3ED3@

and the estimated INCs for the neighbourhood belonging and social cohesion subscales are 0.064 (95% CI 0.058 to 0.070) and 0.010 (0.010 to 0.011) respectively. Figure 5 shows the corresponding estimated reliabilities for the two subscales, accounting for the uncertainty due to both parameter estimation and variability in the number of individuals sampled within EDs. For neighbourhood belonging, the modal reliability is around 0.7, while for social cohesion it lies around 0.3.

That the INCs are so modest is unsurprising given the magnitude of variability at the individual level, and it should be noted that the use of area-level summary measures will depend substantially on the individuals sampled within that area. The estimated reliabilities suggest that, given a sufficient sample size, it is indeed possible to estimate area-level effects based on individual responses; our results suggest this is better for the neighbourhood belonging subscale than for its social cohesion counterpart. However, the social cohesion items have a larger spread, and thus both subscales have advantageous features.

## Discussion

We have taken an adapted version of the Neighbourhood Cohesion scale and undertaken an ecometric analysis of population survey data collected from the socially diverse county borough of Caerphilly. The factor analysis found that the scale distinguishes between two different constructs of neighbourhood social capital: 'neighbourhood belonging', relating to individuals' degree of attachment to their neighbourhood, and 'social cohesion', relating to what people do within their neighbourhood in visiting, sharing favours and trust. The ecometric analysis showed, firstly, that it was possible to reliably measure neighbourhood-level effects [17] and secondly that between-area variability was relatively small. Despite the small INCs, the estimated area-level random effects are still acceptable as measures of neighbourhood cohesion – small INCs and significant parameter estimates are a common finding in multilevel research [1,39,40].

### Previous studies using the Neighbourhood Cohesion scale

Different combinations of the scale items have been included in previous UK studies. Gatrell *et al*. [13] used 11 of the 15 items to derive a measure of 'neighbourhood connections' (items 2, 3, 4, 6, 7, 8, 10, 11, 13, and 14 in Table 1), and one item to measure 'participation or willingness to engage in local social action' (item 9). These subscales were determined *a priori *with no attempt at assessing their ecometric properties, and they were not used at the contextual level. A study set in four neighbourhoods in the City of Glasgow used the 17-item Canadian version [12] of the scale to investigate associations between neighbourhood cohesion, socio-demographic factors and health outcomes, all measured at the level of the individual [14]. The ecometric properties of the scale in this population were not investigated.

Two UK studies have investigated neighbourhood social capital using data from the BHPS [15,16]. The first wave of the BHPS was carried out in 1991 and is an annual survey of more than 5000 households in England, Wales and Scotland (south of the Caledonian canal), sampled using a two-stage stratified cluster design with unit postcodes as the primary sampling units [42]. The BHPS is available to researchers, geographically referenced by electoral ward, within which enumeration districts are nested. All household members aged 16 years and over are interviewed at each wave. Wave 8 included eight items from the original Neighbourhood Cohesion scale (question items 2, 4, 6, 8, 9, 10, 11, and 13 in Table 1). In the first paper, these eight questions were interpreted as a measure of 'social organisational processes' [15]. The reliability of responses was reported using Cronbach's alpha values of 0.83 for men and 0.82 for women. Multilevel modelling was used to quantify the between-ward and within-ward random variance, using the ward as a proxy for neighbourhood. In the null models the INC was 0.212 for men and 0.255 for women. The INCs were 0.116 and 0.136, respectively, for models which adjusted for a range of individual-level socio-demographic covariates. Although these INCs for the shorter eight-item scale are substantively higher than in our current study, no ecometric analysis was carried out to include the between-item variability [15]. The second study using wave 8 of the BHPS labelled the items as 'neighbourhood attachment' and reported Cronbach's alpha of 0.84. No multilevel or ecometric analysis was carried out and the analyses of neighbourhood attachment and health outcome were done at the level of the individual [16]. In our current study we found that three of the eight items asked in the BHPS loaded onto our neighbourhood belonging subscale (question items 2, 10, 11 in Table 1) and five loaded onto our social cohesion subscale (question items 4, 6, 8, 9, 13 in Table 1).

In summary, different studies have used different combinations of question-items from Buckner's original scale, but none of the studies have presented a full analysis of the reliability of the adapted scale in the particular study setting. One study [15] has shown that an 8-item scale could be an acceptable measure of contextual neighbourhood cohesion, attachment or social organisation (depending on the label chosen), but our study is the first to show the uses and limitations of the scale in an ecometric analysis.

### Methodological issues

Any cross-sectional study may be subject to non-response bias. Our dataset is representative of the wider population, based on the similarity of socio-demographic frequencies recorded in the survey to equivalent questions asked in the 2001 census. It is, of course, possible that there may be differences between responders and non-responders in those variables for which there are no available comparators.

In such a socially diverse area as Caerphilly, it is crucial that the sample is large enough to identify area effects. We have shown that this is possible given the available data, though even with a large sample the reliability for the social cohesion subscale is small. One feature of a large dataset is the difficulty inherent in data exploration; with over 11,000 individuals it is nearly impossible to identify individual outliers, data errors and anomalies, and to visualise the structures and patterns in the raw data. We are therefore critically dependent on the model we select to draw conclusions from the data. It is our hope that, building on the pioneering work of the R and S-PLUS [43] development teams, even more powerful exploratory tools will become available to investigate the patterns present in large hierarchically structured datasets.

Given this dependence, it is vital that our chosen model is as flexible and realistic as possible. We are, to our knowledge, the first to combine the method of Raudenbush and Sampson [17] with an ordinal response model. We do so because both the multilevel and ordinal aspects of the model are important; without the former we introduce spurious precision to our conclusions by assuming independence where it does not exist, and without the latter we waste information by dichotomising the more informative five-point scale. We can therefore place more trust not only in the parameter estimates arising from our model, but also in the strength of our conclusions. We believe it is important to *allow *for the possibility of area-level effects, even if – as in the present study – they are only modest.

We have already mentioned that the factor analysis described in this paper is based on the convenient assumptions that individuals gave rise to independent, continuous outcomes. These are not satisfied in practice, and therefore we investigated alternative perspectives. Erroneously assuming independence tends to result in underestimation of variances; since the precisions of factor loadings usually go unreported, this was a minor problem. Of more relevance is the potential for different factor structures to be operating at the different levels of the model, but this does not appear to be the case in our particular dataset. Finally, treating ordinal outcomes as interval variables is likely to be problematic if the hypothetical mapping from an underlying, continuous variable to the observed ordinal quantity is far from linear. In the present application there is no evidence for this, as the intervals between estimated baseline parameters (*θ*_1_,...,*θ*_4_) are fairly regular.

Our ultimate goal in deriving neighbourhood belonging and social cohesion subscales is to use them as area-level covariates in future studies explaining the variability present in measures of individual health. An immediate note of caution is that individuals within a single area may be very heterogeneous in their responses to subscale items, making it difficult to estimate an area-level score. Second, the differences between areas are small relative to variation within them, exacerbating difficulties in discriminating between areas.

Nonetheless, there are also two distinctly positive results from our analysis. Covariates seem to explain only a small portion of the variability present in item response. These item responses could therefore be seen as quantifying something not easily measured by the covariates; that is, 'neighbourhood cohesion'. Also, the distinctivenesses of the behaviour patterns associated with the subscale items vary substantially, meaning that the items could be used for fine discrimination. There are items with which only the most neighbourly of individuals will agree strongly, allowing us to distinguish between 'fairly' and 'very' neighbourly persons. Similarly, there are items with which almost everyone could agree, allowing identification of extremely uncohesive individuals and areas.

In a different population, and using different question items, Stafford *et al*. [44] also attempted to determine the ecometric properties of subscales under the umbrella of neighbourhood cohesion. Like us, they found some subscale scores harder to estimate reliably than others, with reliabilities comparable to the current study. However, two important differences should be noted. First, Stafford *et al*. used binary responses, while our approach calls upon ordinal data and is therefore potentially more informative. In our view using binary responses is neither uniformly weaker or stronger than our approach; as we discuss below there are interpretative difficulties associated with ordinal data. Secondly, and more importantly, Stafford *et al*. do not provide any equivalent of Figures 3 and 4, and thus make it hard to judge the suitability of the question items themselves for discriminating between individuals.

Several studies [45-47] have used large-area measures of social capital in studying its impacts on health, while others have preferred small-area data [48-50]. Of these studies, several [47,49,50] used different scales measuring facets of social capital as area-level measures in multilevel analyses of health outcomes. Our results suggest that this can be done, with caution, using the Buckner scale, after adjusting for individual neighbourhood cohesion scores. A further advantage of the multilevel approach is that we may estimate area-level random effects which are – in the sense of model (2) – independent of the individual-level effects within them. The benefit of using these estimated random effects as area-level measures of neighbourhood cohesion is that their marginal distribution is continuous and assumed to be Gaussian. Continuity of the random effects is advantageous since the area-level measure is then easily interpreted when included as a covariate in other regression models. However, contextual measures may also be determined by aggregation of the responses to the different scale items, having the clear advantage of simplicity of computation. The disadvantage of this approach is that, since scale items are not measurements, there is no guarantee that a person scoring 24 (say) on an aggregate scale by way of four item scores of 1 and four item scores of 5 is at all comparable to another individual scoring 24 with all eight items scored as 3. This is an area where more methodological evaluation is required.

The analyst interested in relating health to neighbourhood cohesion must therefore decide if the latter is truly an area-level phenomenon. If area-level heterogeneity is being masked by random individual deviations, this might be compensated for by making the items more specific. "I visit my friends in their homes" is open to much interpretation about regularity of visits; "I visit my friends in their homes more than once a month" is rather less general, and can either be true or false. Modifications such as this could collapse the dual levels associated with the individual, as both assessor and assessed, down to just one. Strong agreement might mean very different things to different people; it is less likely that 'true' and 'false' do so. Clearly, further research in this field is necessary.

## Conclusion

In this paper, we have applied the ideas of ecometrics to ordinal responses in a hiearchichally structured dataset. Though more complicated than single-level analyses, freely available software exists for exploring and analysing this kind of data. In our view, this methodology should be used whenever interest lies in area-level phenomena which cannot be measured or observed directly.

Greater differences were found within neighbourhoods than were found between them. Large sample sizes, of the order of those used in the Caerphilly Health & Social Needs Study, were therefore needed to discriminate among neighbourhoods. We caution that this is likely to be the case in future studies of area-level social effects. There is, however, cause for optimism about the scale items themselves, which seem indeed to quantify something unmeasured by individual-level covariates – 'neighbourhood cohesion' – and to do so very well.

## Competing interests

The author(s) declare that they have no competing interests.

## Authors' contributions

DLF is the principal investigator of the Caerphilly Health & Social Needs Study. DLF and DMF conceived and carried out the analysis in this paper; FD provided statistical advice. DLF and DMF drafted the paper. All authors read, critically revised and approved the final manuscript.
